# On the typology and the worship status of sacred trees with a special reference to the Middle East

**DOI:** 10.1186/1746-4269-2-26

**Published:** 2006-05-15

**Authors:** Amots Dafni

**Affiliations:** 1 Institute of Evolution, Haifa University, Haifa 31905, Israel

## Abstract

This article contains the reasons for the establishment of sacred trees in Israel based on a field study. It includes 97 interviews with Muslim and Druze informants. While Muslims (Arabs and Bedouins) consider sacred trees especially as an abode of righteous figures' (Wellis') souls or as having a connection to their graves, the Druze relate sacred trees especially to the events or deeds in the lives of prophets and religious leaders. A literary review shows the existence of 24 known reasons for the establishment of sacred trees worldwide, 11 of which are known in Israel one of these is reported here for the first time. We found different trends in monotheistic and polytheistic religions concerning their current worship of sacred trees.

## Background

Frese and Gray [[1]: 26] write: "Trees are a form of nature that represent life and the sacred continuity of the spiritual, cosmic, and physical worlds. A tree is often used to symbolize a deity or other sacred beings, or it may stand for what is sacred in general... Trees represent certain deities or ancestors, serve as mediators or as a link to the religious realm, and are associated with cultural beliefs in heaven or the afterlife... Through association with particular religious or historical events, an individual tree or species of tree acquires the symbolic significance of the events as part of its meaning. A society's religious beliefs about the kinds of trees that are sacred generally depend on the nature and number of trees found in the territory. If trees are plentiful, the forest as a whole will also be an important part of the religion's spiritual beliefs and rituals".

Hughes and Chandran [[2]:78] have already noticed that sacred groves developed originally in traditional societies, which considered themselves linked in a web of spiritual relationships with their biophysical environments. Trees have always been regarded as the first temple of the gods, and sacred groves as their first place of worship; they were held in the utmost reverence [[3]:12.2.; [4]:471; [5]:203; [6]:190; [7]:45; [8], I: 87]. Sacred individual trees and groups of trees characterized almost every culture and religion where trees were capable of growing [[9]:4; [4]:467; [10], I: 109–135, [11]:414; [12]:57; [13]:30]. Thus, it is not surprising to find traces of tree worship in the Middle East [[14]:187]. There is even evidence about the magnitude of tree worship in Palestine in the 19^th ^century "yet in no country are the people more awed by trees than in Palestine" [[15]:54].

It is recognized that trees are not worshipped for themselves but for what is revealed through them, what is implied and signified [[16]:268; [17]:28], especially some kind of power that they express [[18]:35; [19]:359; [12]:57] or their being the abode of supreme beings [[20]:91]. Eliade [[21]:149] noted, "The image of the tree was not chosen only to symbolize the cosmos but also to express life, youth, immortality, wisdom. In other words, the tree came to express everything that religious man regarded as *pre-eminently real and sacred*" (italics in the original). It seems that worship of trees began, among other reasons, because of their long life. In most ancient civilizations trees, whose life-span is several times greater that man's, were treated with the same respect as the elders among men Several generations lived in the shade of the same venerable tree, almost as if it were eternal [[22]:276].

Strangely enough, despite the long list of specific books devoted solely to tree worship [[23]:9; [24]:25; [20]:26; [27]:28; [29]:6], we were unable to find any definition of a "sacred tree". Several authors recognize some "categories" of sacred trees (e.g., [[30]:448; [27]:5–20; 12:58–67]), some of which are not mutually exclusive. To clarify the "hallowed" status of trees, in Israel, we must elucidate the conceptual difference between "blessed", "sacred", and "holy" trees. The difference is not merely semantic but reflects the religious attitude to the adoration of trees. According to the Druze religion only people like prophets could be "sacred"; physical objects like trees may be regarded only as "blessed" [31]. A plant species all of whose specimens are worshipped owing to religious tradition (regardless the exact background) has to be treated as "holy". Simoons [1998:293] distinguishes tree rituals wherein a certain species of tree is considered "holy", as in the case of the sacred fig (Bo tree, *Ficus religiosa *L.), from rituals in which individual trees are "sacred" because of special characteristics or have won respect through their location in a holy place or their association with a holy person (see also [[33]:150] for a similar distinction).

The literature survey shows that the definitions of "sacred tree/wood/grove/forest" are by no means mutually exclusive due to the complexity of reasons for, and the history of, the sanctification of the individual tree, a tree species, or a group of trees. Several authors supply definitions of sacred forest/grove/wood: for example, "... a sacred grove is a stand of trees in a religious context" [[34]:1]; " Sacred trees... describe individual trees or woods which were treated with a certain reverence which, normally, protected them from a wilful damage "[[35]:16]; "Clusters of forest vegetation that honour a deity, provide sanctuary for spirits, remind present generations of ancestors or protect a sanctified place from exploitation. They are treated as sacred by virtue of their location, cultural meaning and history", [[36]:30]; "Sacred grove is a patch of forest or natural vegetation protected and managed by the community considering it to be the reside place of the deities or ancestral spirits" [[37]:2]; for similar definitions see also [[38]:225; [40]:49]; "Sacred groves are more or less patches of climax vegetation... preserved on a religious ground" ([[41]:272] see also [[42]:1063 ; [43]:1204; [44]:1541–1542]). Freeman [[45]:262]) criticized these "ecological" definitions because they were derived from a botanical ideal (climax) and not based on local understanding; his definition is "a piece of garden or forest land... that is dedicated for the exclusive use of particular deities".

Hughes and Chandran [[2]:69] supply a comprehensive definition for a sacred grove as follows: "Segments of landscape containing trees and other forms of life and geographical features that are delimited and protected by human activities believing that preserving such a patch of vegetation in relatively undisturbed state is necessary for expressing one's relation to the divine or to nature".

Gupta [[20]:19] distinguishes a "tree-god", whose worship became organized into a definite religion, from a "tree spirit", whose propitiation degraded the level of sorcery and incarnation. In practice it is impossible to discern, "spirits", "demons", and "jinns" (general supernatural agents) as against "goddesses, "gods", and "the deity" (religiously established worshipped elements). In the Middle East and North Africa, specific trees may be considered the abode of jinns, demons, or spirits, but these supernatural powers are never worshipped as a kind of "god". No religious ceremonies are associated with or performed near these trees; these are regarded as heathen rites and are strictly prohibited.

The various definitions of "sacred tree/grove/wood/forest" may be classified according to four groups of criteria: **A. **natural elements: the physical characters of the tree; **B. **supernatural elements believed to reside in the tree and act upon humans. **C**. human ritual behaviours related to the trees and **D**. botanical criteria such as climax and high biodiversity. A sacred tree/grove/wood/forest may contain the following seven elements: 1. It is the abode of a supernatural power. 2. It is well delineated physically/geographically. 3. The trees are protected by taboos from cutting/exploitation/disrespect/secular behaviours. 4. It is related to historical/cultural/religious issues. 5. The area is protected to please the supernatural powers so as to ensure their benevolence or to avert their malevolent power. 6. It is a piece of natural vegetation (in most cases). 7. It is a ubiquitous phenomenon not limited to any specific religion or geographic territory.

As a practical working definition we suggest treating "sacred trees" as "trees that are subjected to practical manifestations of worship, adoration, and/or veneration that are not practised with ordinary trees". These trees could be single units, groves, forests, or all the specimens of a certain botanical species.

Many religions relate to "metaphysical" trees such as "cosmic tree", "sky tree", "inverted tree", "tree of life", "celestial trees", " tree of wisdom", and "tree of knowledge". These "types" are not mutually exclusive [[6]:273–278; [1]:27–28, [[9]:1–23] Some of these "spiritual trees" are identified with specific species: The Indo-European cosmic and tree of life with oak [[6]:278], the Indian "sky tree" with *Ficus religiosa *[[1]:27], the Assyrian tree of life with date palm [[46]:8–13; [47]:132–133]; while the Egyptian "tree of life" is identified as a date or as sycamore (*Ficus sycamorus*) [[48]:76]. Belief in "metaphysical trees" is not necessarily evidence of practical tree worship [[6]:272].

In this study we exclude "metaphysical trees", while taking into consideration that sometimes the boundary between these trees and reality is not clearly delineated. The present paper is an attempt to elucidate which causes of tree sanctification are characteristic of the Middle East as against other regions, and if differences exist among ethnic groups in Israel on this matter.

## Methodology

The field study (1999–2005) centred on Arab, Bedouin and Druze villages in Galilee. Informants were asked about the reasons why certain trees became sacred. The survey covered 97 informants, consisting of 38 Druze, 59 Muslims (36 Arabs and 23 Bedouins). We consider "Arabs" people who have been settled in their villages for several centuries, "Bedouins" people who originated from the deserts of Israel and Jordan, migrated to the Galilee at the last three centuries and were nomadic till the end of the 20^th ^century [[49]:30]. The Druze are an East Mediterranean group adhering to a religion that was established in Egypt the 11^th ^century [[50]:3]. Today they are concentrated in Lebanon Syria and Israel [[50]:8–14]. The belief in the revelation of God in the form of a human being is considered the most important fundamental principle of the Druze faith [[50]:15]. Druze faith is not ritual-ceremonial religion in essence, but rather a neo-platonic philosophy [[9]:17].

We distinguish "Arabs" and "Bedouins" in attempt to discern different traditions regarded sacred trees which may reflect the different origin of nomads and settled village people. The survey excluded Christians, who hardly believe in sacred trees, while in the Jewish sector the adoration of trees is a new trend of the last two decades and almost all the worshiped trees are already known as old Muslim sacred ones in the vicinity of graves of supposed historical Jewish righteous personalities.

In each village we made a preliminary survey to locate the knowledgeable people in advance and we also approached important religious leaders to examine theirs attitudes to the veneration of sacred trees, then informants were chosen according to their knowledge of common traditions and/or religious status. The average age of the informants was 57.7 (+/-14.8) years. Respondents were 95 males and two females (in general women are reluctant to be interviewed, and when they agreed the interview was held in the presence of other family members). Because of the refusal of most of the informants to be videotaped or recorded all the study is based on oral interviews and field notes that were taken on the spot. The interviewees were asked, "Why have specific trees [especially in their home village] become sacred?" and "How is al-Khader [= Elijah] related to trees?" this question was introduced because this prophet is the most popular one (see below) and at not less that 30 sacred trees are named in his honour [50, Dafni, unpubl.].

## Results

The answers for the question "Why are trees venerated?" are presented in Table 1

**Table 1:** Reasons for sanctification of trees Numbers indicate the percentage of informants in each ethnic group that gave a certain reason.

Reason	Druze	Arab	Bedouin	**References from Palestine**	**Other regions (selected references).**
1. Tree is the abode of a Welli's/saint's spirit.	0	58.3	52.0	[60:303,331;55:91; 57:264; 56:152; 106:89]	[Iraq (107:92); Iran (87,I:375;108:317); Turkey (65:41)]
2. A saint is buried near/under the tree.	2.6	52.7	21.7	[55:93, 61:27, 62:242]	[Morocco (59:67; Turkey (58:227); Algeria (109:311); Turkey (110:215)]
3. Religious and social meetings take place under the tree; well-known figures are associated with the tree.	71.0	44.4	30.4	[56:71; 111:36]	[Ancient Assyria (112:42); Iran (92:142); Morocco (59:68); India (73:19–28)]
4. The tree is dedicated to a prophet.	47.3	22.2	34.7	[56:65]	
5. The species is religiously blessed.	26.3	19.4	17.3	[31]	
6. The tree/forest commemorates events in the lives of saints, heroes, kings, in the tribe's history etc..	36.8	41.6	26.0	[106:51; 55:93; 113:95]	[Assyria (112:42); Ancient Britain (26:44,104); Ireland (35, 19, 21, 25, 33, 35:29, 32, 36); England (118:184); Morocco (114:27); Mozambique (103:229); Uganda (115:37); Ghana (38:225–232;120:366; 121:41); Iran (87, I: 372, 92:142); India (116:26); Nepal; (119:248; 122:335); Okinawa (96:57); New Zealand (117:220)]
7. The tree shows the way to a sacred place.	92.0	0	0		
8. The tree sprouted from saints' staffs.	7.8	11.1	4.3	[56:27; 124:407; 125:378–379]	[England (26:21); Ireland (35:35, 38, 40); Poland (6:55); Morocco (59:68); Iran (125:41); India (126 (127:287 in 21:27); New Zealand (128:68)]
9. Tree provides shade in the desert	0	0	17.3		[Ancient Egypt (129:121); Iran (87,I:373); India (130:43)]
10. The Tree Grows/is planted over the grave of the saint.	2.6	2.7	0	[106:89]	[Ancient Celts (5:202); Egypt (66:56); Morocco (63:80); Zanzibar (131:36); Mongolia (79:2830]
11. The tree is sacred because of the tree's size, age, shape; being evergreen or having a strange form.	0	5.5	0		[Ancient Greece (76:26; 34:10); Ancient Rome (3, 12.2.3.; 132:67); Pagan Europe (74:38; 132:67); Russia (133, I:194); Siberia (141:58); Armenia (135:320); East Africa (78:4); Kenya (139:151); Cameroon (140:100); Sierra Leone (136:47); Iran (92:142); Inner Mongolia (80:279,283); India (137:239); China (138:759); Taiwan (134:5,III,1)]
12. The tree has healing powers.	0	0	0		[Britain (26:44); Uganda (143, II: 832); China (142:412–413)]
13. The tree is the abode of supernatural beings: tree spirits, djinns, demons, deities, goddess, angels, divine beings, dragon, monsters, etc.,	0	2.7	0		Ancient Egypt (151:89; 70:40); Minoan (152:141); Ancient Greece (34:10, 16, 20); Ancient Celts (5:198); Old Scandinavia (6:52); Teutonic mythology (8,I:71); East Africa (78:4); Uganda (115:37); Zimbabwe (101:6); India (98:9,15; 137:240,242); Ethiopia (154:3); Zanzibar (131:35); Mozambique, 103:229); Sierra Leone (156:102); Nigeria (157:292); West Africa (158:44); East Africa (78:4); Central Africa (159:317); Siberia (155:117); Iran (144:125); Inner Mongolia (80:277); Nepal (150:334); India (137:242; 40:280; 43:1578; 153:47); Thailand (147:330,147:66); China (138:759;161:131); Japan (145:34,35; 60:176); Indonesia (148:317);Okinawa (96:46,57,63;149:177);Micronesia (102:9); Papua (162:72)]
14. All the individual trees of the species are religiously sacred.	0	0	0		[Dahomey (172:54); India (165); Armenia (166:5); Tibet (168:325); China (138:759); Micronesia (167:13); Kiribati Pacific Islands (102:46); Chile (169:146); American Indians (164:121)]
15. To commemorate miracles occurred near the tree or related to a saint.	0	0	0	[(56:70]	[Ireland (35:37,43); Egypt (90:17); ]Morocco (59:67,259); Iran (108:317); India (137:243); Tibet (171:276); Taiwan (134:5,III,1); China (172:133)]
16. Tree is abode of ancestors' souls.	0	0	0		[Old Celts (5:103); Mozambique (103:231); Ghana (121:41); Madagascar (176:19,20, 177:61); Zimbabwe(101:6,9;126, :378); Sierra Leone (136:47); S. Rhodesia, (178:102); Ghana (120:366; 179:149, 18:35); Kenya (180:135; French Guinea (181:14); Inner Mongolia (80:277,280); India (137:242; 173:332; 38:593); Laos (174:4; 182:7); Indonesia (175:310,318); Papua (162:72); Australia (183:163)]
17. The tree is located near holy water source.	0	0	0		[Ancient Greece (34:20,25,34); Ancient Syria (82:115); Ireland (35:40–42; 83:30); Egypt (90:17); Algeria (184:72); Tanganyika (185:39); Turkey (110:215); China (138:759)]
18. Tree/grove is grown/planted in sacred places, burial sites, graveyards, temple worship sites.	0	0	0		[Ireland (35:29,44); East Africa (189:414,415,); Ghana (120:366;121:41); Kenya (180:134); Madagascar (191:83); Central Asia (190:99); Sri-lanka (150:176); Nepal (151:270,334); India (137:242; 32:293; 188:105;192:433; 193:237); Japan (186:180, 185:133) ; Taiwan (134:5, III, 1); China (161:131; 194:704)]
19. The tree is useful.	0	0	0	Sinai (195:184)	[Ancient Assyria (112:42); Europe (5:199; 6:149); Ethiopia (154:4); Nigeria (157:292); S. Rhodesia (178:126–127); India (137:252, 196:157, 196:400; 73:21); Himalaya (38:58)]
20. Tree creates a religious atmosphere	0	0	0		[Ancient Rome (196:IV, 12, 3: 6:52); India (201:67; 199:153, 1981:281)]
21. The tree attracts lightning	0	0	0		[Europe (202:676; 203:79; 6:161); India (137:239); American Indians (204:15)]
22. God is transformed into a tree.	0	0	0		[Ancient Egypt (205: 15.16; 14:191); Russia (100:700)]
23. The grove/forest declared sacred to insure the community resources/water catchments	0	0	0		[Uganda(115:38);Mozambique (207:12);India ([206:8); China (163:131)]
24. Planting a tree in high places as an abode for gods	0	0	0		Mongolia [79:282].

When the interviewees were asked "why trees are venerated", they gave the following answers (the bold numbers are related to certain informants, see Appendix):

1."The sacredness of the tree originated only from a sacred tomb or the place of a Welli" (23 infor-mants).

2."A man that prays to a tree is a heathen; we need to pray only to God. The tree is temporal, only God is eternal. If the sacred tree is not able to protect itself against being cutting down, how will it protect humans? We have to pray to God, who is the Creator. We have to worship God, not trees" (**1**).

3."We have to believe in God, not in trees; it is against the religious law" (**2**).

4."God protects sacred trees" (**3**).

5."Sanctity rests only on prophets; it is forbidden to sanctify objects like stones and trees" (**4**).

6."People have prayed to trees since Roman times, and [the tendency] remains inside the human. I believe only in one God" (**5**).

7." The sacred tree is a path between man and god" (**6**).

8. "The importance of the sacred trees depends on the saint; the holier the man, the holier is the tree " (**7**).

9."Sacred trees are the memento of a saint or holy man, so their importance is relative to the man's holiness" (**8**).

10." The blessed tree is a symbol of the prophet, not an object per se" (**9**).

11."Holy is that which is sanctified by God, blessed it is by humans'" (**10**).

12."Each blessed tree has an angel or jinn or demon that resides inside and protects it" (**11**).

13."Religious people give the tree their sacredness; maybe the saint planted the tree or was buried underneath it" (**12**).

14."Holiness is accorded only to the prophets and it is forbidden to sanctify objects like trees and stones" (**13**).

15."Faith gives the strength and a tree near a sacred grave gives blessing" (**14**,**15**).

16."In our religion (Druze) we don't sanctify people or trees, only God" (**15**). "We love the Prophet but we don't sanctify him" (**16**).

17."Blessed trees are memorials to singular figures in Druze history and religion; because the Druze tradition forbids any tomb sign or offerings, special people are remembered by large blessed trees" (**17**).

18."The man (a religious figure) is sacred and the tree is blessed. The tree belongs to the people and the saints are the prophets and God". "The blessed trees are a monument to special figures in the history of the Druze religion. In our religion there are no gravestones or offerings in graveyards; the trees commemorate the deeds of these special personalities" (**11**).

When our informants were asked, "How is al-Khader related to trees?" we received the following answers:

1. "Every tree that is dedicated to al-Khader is a blessing; he is even closer to God than Nabi Shu'ayb (the Druze's most important prophet)" (**15**).

2. " Al-Khader has the power of 70 prophets" (**18**).

3. "Every place where al-Khader sat became green" (12 informants).

4. "The place is sacred and green because al-Khader rested there" (11 informants).

5. "The colour of al-Khader is green" (21 informants).

6. "al-Khaher had the habit of sitting under trees", "al-Khader is related to trees", or "al-Khader loves trees" (12 informants).

Based on Table 1, the analysis of the interviews, additional field observations and the literature survey we find the following trends: (figures in parentheses are the numbers as in Table 1).

1. The most common reasons for the sanctity of a tree are its being the abode of a saint's (Welli's) spirit and the location in its vicinity being the grave or shrine of a saint ("Makam"); these two reasons were strictly confined to Muslims and were never given by Druze.

2. Dedication to a prophet, the most popular of whom is al-Khader.

3. Religious and social meetings: The most common reason given was that the tree was a place beneath which religious leaders used to gather for preaching/discussions/meetings/court sessions/judgements. This reason was more represented among Druze than among Muslims.

4. Events in lives of saints. These may include praying, preaching, resting, living or visiting under the specific tree. This reason seemed similarly represented among the three ethnic groups.

5. The tree species is adored because of a religious dedication: the only tree that in this category was *Ziziphus spina christi *(see [31] and below).

6. The tree shows the way to a sacred place: this reason was given only by Druze, all of whom

mentioned one specific tree (see below).

7. Some reasons (13, 14, 16, 18) which seem to be connected especially to polytheism are absent from the Middle East.

8. Some reasons that are known from the classical world, as well as from pagan Europe, are very rare or not known today in the Middle East (10,11,13,14,15,16,17,19,20,21,22).

9. All the recorded reasons (except "showing the way to a sacred place") are already mentioned in the literature especially in ancient Europe, North Africa, and the Fertile Crescent.

10. Some reasons, as far as the author is aware, have never been recorded in the Middle East: (12–14, 16–18, 20–24).

### New and abandoned sacred trees

During our study we came upon four cases in which the "sacredness" of a tree could be declared "de novo" or annulled for political as well as religious reasons.

On the Jewish Day of Atonement in 2003 several people from the Druze village of Dalyat al-Karmel (Mt. Carmel) decided to annex an area that was in dispute with Israel's Nature Reserve Authority. In an organized operation an asphalt road was paved and a large oak tree (*Quercus calliprinos*) in the at Nahal Alon Nature reserve, was declared blessed and a new religious building ("Khalva") was erected at this place. By this act the authorities were forced to accept the Druze claim to the area (**19, 20**). After the religious ceremony the local people accepted the tree as blessed and since that day they regularly place put rags and flags on it, and honour the tree as is customary with every "sacred tree". The action was "covered" by a religious tradition (**21**). The local keeper of the "Khalva" related that a note dating back 180 years was found in one of the holy books. It told of a pious virgin, "Sit Khadra", (the green lady) who lived in the area and was famous as a successful farmer. After this note was found it was decided to sanctify the area in which she was lived to commemorate her (**22**).

A case of withdrawal of sanctification occurred in the town of 'Arrābe (Lower Galilee). There stands a very famous ancient tree of *Pistacia atlantica*, which is "the" sacred tree of the village. It is named "the tree of the Saddik (righteous man)" believed buried under it. In 1961 an archaeological excavation discovered that this was a Jewish grave (the burial direction was north-south, not west-south as in the Arab tradition). The authorities constructed an old-style building and named it officially after "Khanina Ben Dossa". A famous Talmudic sage (the tradition concerning this burial place is dated to the 10^th ^century [[52]:324–325; [53]:152–155]. From the time the tomb was declared Jewish, the appeal of the tree as sacred to the Muslims declined and fewer people go there to ask the help of the righteous man buried underneath. The various informants (n = 10) held conflicting views as to whether the change was influenced by the local religious leaders.

Similarly, recently (around 2002) religious leaders in the village of Mes'hed (near Nazareth) were unhappy about the veneration by Jewish people of a tree in its midst (a large *Pistacia atlantica *that is a centre for vows and is abundantly visited by local people as a "wishing tree"). Nevertheless, people continue to visit the tree as usual (**23**). In the town of Sakhnin (Lower Galilee) there is an ancient cave containing the tomb of Rabbi Yehoshua D'Sikhnin [[52]:302–303, [[53]:150–151]. The place is renowned throughout the region for its miraculous powers to cure sick people, and especially barren woman. Very close to the cave there is a large sacred Ziziphus *spina christii *tree stands in the middle of an Arab cemetery. In 1980 a local Muslim religious leader decided to forbid the attachment of rags to the tree as well as prayer there, because the place was sacred to the Jews but not the Arabs. The ban held for five or six years, after which the Muslim people returned to their old tradition, saying that the Jewish saint helped them (**24**).

## Discussion

All the known reasons for the establishment of the sacred trees/groves/forests may apparently be sorted into several generalized categories: dedication to supernatural beings/powers, relation to established religious rituals and ceremonies, dedication to people, commemoration of historical or miraculous events and practical, economic and conservational reasons. It is noteworthy that in a single community/religion more than one reason, for the establishment of a sacred grove (and we suggest to extending this to all types of sacred/holy trees) may be established. More than one aspect was caused by a combination of economic, religious, social and environmental factors, to yield of social, environmental, economic as well as religious reasons [[55]:30]. Political as well as religious reasons could lead to the declaration of new "sacred tree" or to the denial of well known existing ones, as was shown in this study.

### Trees as an abode of a saint's spirit

As it can be seen (Table 1) the most common "function" of the sacred trees in the Middle East is to serve as the abode of the spirit/soul of a saint (Welli). Curtiss [[56]:75, 77, 79], regarding the status of saints in the Muslim world, noted "... orthodox Moslems insist that the saints are only mediators that a worshipper asks his Welli to intercede for him with God... These saints are really departed spirits, connected with some particular shrine, chosen because they revealed themselves there in the past, and where they are wont to reveal themselves now to these who seek their favour .... The worship of the saints is like that of the ancient Baalim. They are the deities whom people fear, love, serve and adore". Cannan [[57]:151] held that "The present-day peasant does not venerate the trees themselves but the divine-power which acts in them and which is derived from the godly person whose soul is supposed to be still inhabiting the shrine, tomb, cave or spring with which they have become associated. Often these holy men have appeared either in the tree itself or near by". The objection of the religious leaders, and role of the tree as mediator, were also stressed by our informants.

### Trees and saints' graves

In the Middle East, as in North Africa, a saint's grave is closely connected to a sacred tree; trees beneath which saints are buried are regarded as "sacred trees" [[56]:93]. The identification of the sacred tree with the saint's grave imparts to it the miraculous and magical powers of the holy man [[58]:264; [56]:94; [57]:71; [59]:176–177]. Westermarck [[60]:74] comments that the existence of sacred groves around saints' tombs maybe related to the people's avoidance of cutting down these trees for fear of the saint's retribution, even in places where there is no tomb but only a tradition of the holiness of the location, especially in cases where it is not at all clear who the saint is. Thus it seems (in Morocco) that the worship site exists owing to the grove more that the grove exists owing to the site. Canaan comments that sacred trees that are not connected with graves never bear the name of a specific personality [[6]:70]. Our data show that this is not a rule, and today many sacred trees in Israel are not related to tombs; however, these structures might have disappeared in the course of time. Because tradition relates the tree to a saint, it is respected accordingly [[61]:331; [62]:27–28]. In practice it is impossible to determine which came first, the tree or the grave, because of the customs of burying important people near sacred trees [[63]:242] and of planting trees on saints' graves (Morocco [[64]:80]. Hasluck [[59]:238] concluded on this subject in Turkey, "It is often impossible to say whether the sacredness of these groves is primitive and their connection with saints evolved from it, or whether it is secondary and due to their proximity to saint's graves"; the same situation exists in Syria [[65]:179] (at that time Palestine was a part of "Great Syria").

Zarcone [[66]:41] notes that certain trees are sacred because of their connection to a specific figure in the Islamic tradition, and tree veneration may be the fusion of tree worship in general as a part of the supernatural. So sometimes the tree confers holiness on a specific site or a sheikh's tomb as a part of his hagiography. Blackman [[67]:57] mentioned that (in Egypt) trees were sanctified because they grew at a place where a saint was murdered and they bear his soul. He also argued that this is parallel to the ancient Egyptian myth that the sycamore *(Ficus sycamorus*) grew out of the dead body of Osiris [[68]:29,339].

The conclusion is that it is not clear if the tree became sacred because of the saint, or the personality became sacred because of the tree.

### Social and religious meetings

Especially among the Druze, trees acquired their sacredness through the habit of historical religious leaders meeting under them, to preach or/and to discuss religious issues. When leaders from Lebanon used to visit their faithful in the Galilee they customarily met their local colleagues beneath these trees, which came therefore to be considered "blessed". Sacred trees can't be considered as an abode of a soul because the Druze believe in the transmigration of souls, a person's body is a kind of clothing for the soul, and with the person's demise the soul passes over to the body of a newborn child" [[9]:60]. Thus, souls cannot reside in a tree and graves are not revered, trees were blessed on account of the visits of the religious leader; but souls are never connected with trees.

### Dedication to a prophet

In all the ethnic groups we found sacred trees that were dedicated to a prophet. When we asked specifically who the most common, people mentioned al-Khader, who is highly respected by Muslims as well as by Druze. Not less than 30 places (some of which contain "sacred trees") in the Holy Land are named after him [[69]:13–34]. This prophet is adored by Druze as well by Muslims. Al-Khader (also Al-Khidr or Al-Khudr), who has common features that characterize Elijah and St. George [[8]:48–65], is the most popular of all saints in the Middle East [[56]:84; [70]:288; [59]:319–336]. This prophet is closely connected with sacred trees, as was also found in this study. This notion is revealed in the name: Al-Khader means "the green one" [[70]:288; [69]:9]. It is believed that every place on which Al-Khader sat became green. This concept may explain why so many trees are dedicated specifically to this prophet.

### Religious species ("holy trees")

In many cultures (see Table 1) all the individual trees of certain species are sacred, the most famous being *Ficus religiosa *underneath which Buddha received his enlightenment [[71]:24; [32]:41–100]. The only tree in the Middle East that can be regarded as close to "holy tree" is *Ziziphus spina christi*, which is mentioned in the Quran. Individual trees of this species are highly respected, by Muslims, but are worshipped only in connection with a saintly person, and not *per se*. The Druzes treat this species at the same manner, but it is still regarded as a "blessed" tree [31]. All the other categories of worshipped trees (Table 1) can thus be considered as "sacred trees".

### Events under the tree

In our survey Muslims as well as Druze mentioned events in the life of the saints/prophet/religious figures as one of the main reason for the sanctification of trees. Curtiss [[56]:93] noted that "trees under which saints rested are considered holy". We can add that it was sufficient for the saint to teach, preach, or pray under a tree to make it sacred. In Israel we failed to find even a single sacred tree that commemorates a specific historical event. In Britain, for example, many such cases are found, although it is hard to discern what comes first, the event or the sanctification of the tree [[26]:44,104], see also Table 1 for more eamples.I.

### Showing the way to a sacred place

Near village of Mghar (Lower Galilee), on the main road to Nabi Shu'ayb (believed to be the grave of the prophet Jethro), the holiest place for Druze in Israel [[50]:217–218], there is a huge Christ's Thorn Jujube (*Z. spina christi*) tree. In the past this important tree served as a meeting point for pilgrims before approaching the holy place for the festival of Nabi Sua'yb (on 25 April, every year). Whoever arrived first waited for the others under that tree. Over the years the tradition of the first meeting point took root, and this specific tree became a station for praying as well. It is the only individual tree of his kind that reached the status of a "blessed tree". When the pilgrims reached the tree they became very excited, and this is how the tree came to be named "Sidrat Nebi Shu'ayb" (the Prophet's Jujube) [31].

### Sprouting from a saint's staff

In some places we heard that sacred trees had sprung from staffs carried by saints or religious pleaders. Similar stories are known also from other countries and are not endemic to our region (Table 1).

### Shadow in the desert

This reason was mentioned only by the Bedouins and can be looked as vestiges of old traditions reflecting their history.

### Planting trees in sacred places/groves growing in sacred places and tree's as having healing powers

These reasons are rare in the Middle East and are not common worldwide.

### Tree characters

Only two of our interviewees mentioned tree size as a reason for its sanctification. In the literature (Table 1) large tree size and evergreen-ness are mentioned as important characters that lead to tree veneration. In Israel at least two of the common sacred trees are deciduous (*Quercus ithaburensis *and *Pistacia atlantica*). These two species can grow to a considerable size, and it seems that this is the very reason why they were venerated. These observations are run counter to Wilson [[62]:6] who maintains that all the oaks on saint's graves are evergreen. In one village (Sajur, Upper Galilee) there is a blessed *Styrax officinalis *tree; the keeper of the tree is convinced that this specific tree is Venerated (named al-Mubarakeh, meaning "the blessed") because it is said that it is the only evergreen individual of this deciduous species (**24, **it is a well known story in the village, n = 12). Our observation failed to corroborate this, although it is in a protected garden and the leaf fall is somewhat shorter in comparison with other *Styrax *trees growing in an exposed habitat. This case brings to mind a sacred platanus (*Platanus orientalis) *in Gortyna that was sanctified in Ancient Greece because it was an individual evergreen plant (the species is generally deciduous) and connected with the abduction of Europa [[3]: 7.1; [72]:176].

Huge trees were objects of veneration and a manifestation of the Almighty [[23]:2; [73]:29]. The most famous "great tree" is the oak, which is the foremost tree in European mythologies and tree worship [75]; [76]:188–191; [77]:23]. In the words of Folkrad [[77]:21] "The Oak, the strongest of all trees, has been revered as the emblem of the Supreme Being by almost all the nations of heathendom" Porteous [[6]:150] explains why these trees were venerated: "As year after year passed with the same continual changefulness, trees, or perhaps one outstanding tree on account of its size and age, would come to be regarded with a special reverence, and primitive imagination would people it with all sorts of beings, such as Gods, Nymphs, and Demons". The Kikuyus in East Africa select large trees for veneration. A sacred tree must be high because it is deemed nearer to the god as a medium through which prayers are to ascend [[78]:4]. Sometimes the reason for sanctity is the strange or the unusual appearance of the tree rather it size [Japan [79:188; Inner Mongolia [[80]:279,283].

### Trees as an abode of super natural beings

Trees that are the abode of deities, gods and ancestor' souls were never recognized in the Middle East, and it seems that these reasons are confined today to polytheistic religions. The recurrent pattern of this kind of sacred tree/forest/grove is highly typical: the tree/grove/forest is dedicated to a certain god/deity or ancestral soul, which is in charge of the welfare and well being of the community/village. To ensure supernatural blessing, certain rituals must be performed to please the god or the ancestors to win their favour [[80]:1578; [18]:35; [81]:350–351].

### The tree is located near holy water source

The survey of sacred trees in Israel ([77] and our observations) revealed that they are rarely adjacent/related to water sources; we were unable to find any evidence that this vicinity is the reason for the sanctification. In ancient Greece [[34]:20, 25, 34] sacred trees were sometimes associated with holy water sources. Concerning ancient Syria White states, "this early nature worship, whether of numerous Baalim of the Syrian oases or the local nymphs of the sacred springs of Hellas, required only the marked-off enclosure of holy ground beside the spring, or about the circle of trees in the sacred grove" [[82]:115]. Sacred trees associated with sacred wells are common in Britain even today [[35]:40–42; [83]:30].

## Conclusion

Of the 24 known reasons for the creation of sacred trees/groves/woods/forests 11 reasons (Table 1, reasons 1 to 11) were recorded by us in the field while the other 13 were compiled from the literature and were almost never recorded from the Middle East. Only one reason (showing the way to a sacred place) was never recorded before and was found to be endemic to the Druze and is related to one specific very famous tree. Generally, most of the local reasons seem to be more confined to the monotheistic religions as "vestiges" of the old paganism (see below) and appear very rarely in polytheistic religions. Some of the remaining reasons (especially 13, 16, and 18) are typical of polytheistic religions as a part of the regular rituals and are seldom mentioned in monotheistic areas. The evidence from ancient Europe, Egypt, and the Middle East may reflect the prehistoric pagan heritage that was partly adopted later by the monotheistic faiths. These replaced tree-dwelling spirits, gods, and souls by saint worship performed through the trees. Owing to the long and aggressive struggle of the monotheistic establishments (Jewish, Christian, as well as Muslim) with the old paganism, "tree adoration" became marginal and prevails today mainly in rural areas of the Middle East and North Africa.

In the pre-Islamic pagan world tree worship was quite common [[84]: 169–107; [14]:185; [85]:181]. These trees were worshipped as the abode of jinns and spirits, and were treated as possessing godly characters [[1]:30]. Several authors consider tree veneration in the Muslim world a relic of old heathen worship of tree-spirits or gods, which has survived, in a thinly disguised form, throughout all the ages of Christian and Islamic supremacy [[77]:242; [14]:199; [93]:50; [86]:34; [77]:92]. After the Arab conquest the tree spirits/deities/gods of the early heathen inhabitants were replaced, after the Arab conquest, by the spirits of the Muslim saints, the "Awlia" (= plural of Welli), which may survive and appear in sacred trees (Palestine [[77]:151]; Iran [[87], I:378]; Morocco [[88]:97]). A completely different view is expressed by Albright [[89]:284–286] who considers the Welli cult of Palestine and Syria as merely as a phase of the saint-cult of the Mediterranean region and differing only in detail from the saint-cult of the lower classes in other Mediterranean lands. Moreover, he argues that this saint-cult goes back to the Christian saint-cult of the Eastern Roman Empire in the early Byzantine centuries and is Hellenistic-Roman, not Semitic, in origin. Hornblower [[90]:19] similarly notes, concerning sacred trees in Egypt, that the local gods were replaced by local saints, first Christian and then Muslim. With the crystallization of Islam, these old venerated trees were cut down and this kind of worship was strictly forbidden [[91]:318; [92]:243–244]. The practical result, as can be seen today (Table 1), may be considered a kind of "functional religious replacement": no longer are the trees regarded as the abode of tree-spirits, deities, or gods, as in earlier heathen times, but as the abode of saints, who are regarded as the messengers of God himself. This kind of "softened idolatry" exists to the present day, despite the Islamic regime, which has proved too weak to eliminate it, especially in rural areas. In consequence of our survey, we fully agree with Frazer [[93]:43] who, very pithily, summarizes the status of sacred trees in the Middle East: "Thus the worship at the high places and green trees, which pious Hebrew kings forbade and prophets thundered against thousands of years ago, persists apparently in the same places to this day".

A review of the reasons for the creation of sacred trees/groves (Table 1) shows some kind of dichotomy between the monotheistic legacy of the sacred trees in Europe, the Middle East and North Africa as against the polytheistic traditions in the world. In polytheistic religions, especially in Africa and Asia, people still see the sacred tree/grove as the abode of deities, ancestors' spirits, etc. (see Table 1), which may reflect the old "pagan" customs that prevailed in Europe, the Middle East, and North Africa at the remote past. Fergusson [[94]:62] noted that while all the monotheistic religions fought ancient tree worship, Buddhism elevated it to a higher level of veneration. According to Avasthi [[95]:7] polytheism in India allows multiplicity in worshipping objects like trees, rivers, or the village deity. These objects vary from person to person and festival to festival. The celebration is public whereas the worship of gods and goddesses takes place in the family.

In the polytheistic world the sacred grove/wood it is a centre of common tribal activities. Sometimes access is limited to certain people and/or certain occasions, and the grove is kept by the community or by a special priest, additionally to the general taboo not to harm the tree [Sierra Leone (36:311); Okinawa (96:5,18); East Timor ([97]:224); India ([41]:49; [98]:9; [99]:712); Russia ([100]:699); Zimbabwe ([101]:6); Northern Ghana ([18]:35); Vanatua (Pacific islands, [102]:9); Mozambique ([103]:229); West Africa ([104]:45)]. In the present-day Middle East the sacred tree is a centre for individual ritual behaviour with free access.

There is no doubt that the present-day vestigial tree worship in Europe is a result of "Christianization" of the old pagan religions. Elworthy [[105]:107–108] noted " The remarkable similarity in the customs (of tree veneration) all over Europe points to the conclusion that tree worship was once an important element in the early religion of mankind, especially of the Aryan stock, and that the singular uniformity of the rites and ceremonies which can easily be shown to exist in widely separated countries, fully warrants us in believing that they have not much changed from very remote ages, and that the practices continued down to a very recent period by peasantry.... were substantially identical with the same rites and ceremonies observed by Egyptians, Etruscans, Greeks and Romans". At this point it is fitting to cite Lucas [[35]:34] "At first, in brief, the church came to the [sacred] tree, not the tree to the church". Almost the same view is expressed by Robertson-Smith [[14]:186–187: "The worship of solitary trees survived the fall of the great gods of Semitic heathenism... The solitary tree may in certain cases be the last relic of a ruined heathen sanctuary". Likewise Porteous [[6]:162] comments that "It [tree worship] was... so deeply ingrained in the human hearts that in many cases it was utilized by the Church for its own ends by blessing the most ancient and venerated trees, and by erecting Christian altars and placing crucifixes and images the people had sacrificed to the heathen divinities" (see also [[8]:I,86–87]).

The present survey shows that tree veneration is still quite common in Israel among Muslims and Druze. The reasons for sanctification of trees are mainly connected with the adoration of saints and prophets. While the Muslims connected sacred trees with saints' souls and graves, the Druze, who believe in transmigration of souls, relate the blessed tree mainly to the events and activities of prophets and historical religious leaders.

A worldwide comparison shows the great similarity of the monotheistic religions, in which saint adoration is the main focus for tree worship. In polytheistic religions sacred trees are mainly connected with local gods, spirits, demons and ancestor veneration, none of which is found in the present-day Middle East.

## Declaration of competing interest

The author(s) declares that he has no competing interests.

## Appendix

List of the informants who are cited personally according to theirs appearance order in the text (Bold numbers in the text to differ from the literature sources) the given data are: The name of the informant, his age, ethnic group, place, and the date of the interview.

1. Zaki Abu Bilal Hashad, 67, Muslim imam, Tarsħîha, 19 Dec. 2005

2. Qāsim Bader, 45, Druze, Keeper of the sacred place of Nabi Sabalān sanctuary, 9 June

2002.

3. Faraj Kiblawi, 67, Muslim, Tarshīħa, 19 Dec. 2005.

4. Ħāmed abu Mustafa, 45, Muslin, 'Arrābe, 21. Dec. 2003.

5. Ruqqiya Maghis, 50, Joreih, Bedouin. 27 March 2005.

6. Qāsem Shibli, 32, Bedouin, Shibli 21. Oct. 2004.

7. Sa'īd Muħmmad, 90, Druze, Yānuħ, 25 May 2003

8. Sa'īd Maħamûd Sa'īd, 69, Druze, YānuĦ, 25 May 2003.

9. Akab 'Amashe, 45, Druze Sheikh, Buq' ātha, 12 Dec. 2001.

10. Sheikh Nûr Rifa'īyye, 40, Majdl Krûm, Sufi Muslim, 24. June 2000.

11. Sheikh Šhahīn Ħussein, 70, Druze a religious leader, Beit Jan, 12. Sept. 2000.

12. 'Âdel Abu Ħamid, 57, Muslim, Kufr Manda, 16 April 2004.

13. Ħāmed abu Mustafa, 45, Muslim, 'Arrābe, 31. Dec. 2003.

14. Sa'di Qaramān, 75, Muslim, Damûn, 13 Sept. 2000.

15. Jamīl Abu Rā'id Arafāt, 71, Muslim, Mashhad, 23. Sept. 2004.

16. Sāleh Ħatīb, 50, Mghar, Druze, 18 March 2003.

17. Karmel Na'ama, 52, Muslim, 'Arrābe, 6 June 2004.

18. Sueid Hussein,68, Druze, Peqee'in, 8 Nov. 2005.

19. Sheikh Tawkfīq Amashe, 70, Druze, Mas'ade, 12 Dec. 2001.

20. Salmān Abu Rukan, 55, Druze, 'Isfia, 12 Dec. 2003.

21. Mustafa Ħalāwi, 48, Druze, 'Isfia, 15 Dec. 2003.

22. Maħmud Abu L'Raħman Mar'īn, 67, Muslim, Meshhed, 20 Sep. 2004.

23. Yuval Avidor, 35, Jew, Yodfat. 21 Dec. 2003.

24. Zi'ad Tallāl, Druze, 35, Druze, Sajur, 3 Oct. 2003.
